# Construction of an integrated treatment and management model for psychiatric emergency and intensive care units in a specialized psychiatric hospital: practice of subspecialty development

**DOI:** 10.3389/frhs.2025.1691858

**Published:** 2025-10-27

**Authors:** Yi-chao Wang, Hui Yu, Fu-gang Luo, Hong-mei Wang

**Affiliations:** ^1^ Affiliated Mental Health Center & Hangzhou Seventh People’s Hospital, Zhejiang University School of Medicine, Hangzhou, Zhejiang, China; ^2^ The Fourth People’s Hospital of Aksu Prefecture (Kangning Hospital), Aksu, Xinjiang, China

**Keywords:** psychiatric hospital, psychiatric emergency department, psychiatric intensive care units, integrated treatment and management model, practice of subspecialty development

## Abstract

**Background:**

Psychiatric patients admitted through emergency pathways often presented with severe comorbid physical illnesses, which posed challenges for timely diagnosis and effective management in psychiatric specialty hospitals. To address this gap, our hospital established an integrated model that linked the Psychiatric Emergency Department (PED) with the Psychiatric Intensive Care Unit (PICU), aiming to create a continuous and coordinated emergency–critical care system.

**Description of the model:**

The PED–PICU integrated model was developed through progressive institutional innovations, including the establishment of a dedicated PICU, functional integration of the PED, and the creation of specialized rapid-response centers. This model enabled early identification of critical conditions, seamless transfer between emergency and intensive care, and continuity of treatment until recovery.

**Experience and outcomes:**

In practice, the model improved coordination between emergency and critical care teams, facilitated timely interventions, and ensured that patients with severe psychiatric and medical comorbidities received comprehensive management within a single institutional framework. The integration also strengthened multidisciplinary collaboration and highlighted the unique role of psychiatric specialty hospitals in managing complex emergencies.

**Conclusions:**

The PED–PICU integrated model represented a pioneering and unique practice in psychiatric specialty hospitals. By closing the gaps between emergency stabilization and intensive care, it established a closed-loop system that might serve as a valuable reference for developing similar subspecialties and improving emergency–critical care pathways in mental health services.

## 1 Introduction

In recent years, with the evolving spectrum of psychiatric disorders and the growing demand for managing severe mental illnesses, specialized psychiatric hospitals shifted from a focus solely on psychiatric symptom intervention toward a more comprehensive model of integrated psychiatric and medical management. Among patients with severe mental disorders, the high prevalence of comorbid physical illnesses became a key factor influencing prognosis, prolonging hospital stays, increasing readmission rates, and elevating mortality risk (1–4). Although psychiatric intensive care wards dedicated to managing patients in the acute phase of severe mental illness became increasingly common, their capacity to cope with coexisting severe physical conditions remained limited. Research consistently highlighted that patients with severe mental illness were at disproportionately high risk of adverse outcomes when critical physical illness was present. For example, catatonic stupor in schizophrenia was closely linked to life-threatening medical complications and increased mortality (5), while patients with schizophrenia admitted to intensive care units of general hospitals exhibited higher risks of acute organ dysfunction and in-hospital mortality (6). Similarly, multimorbidity in schizophrenia substantially increased mortality risk (7), while long-term follow-up studies confirmed that physical comorbidity was a major determinant of mortality in this population (8). These findings aligned with large-scale international evidence (1, 2, 4), and a systematic review further demonstrated that psychiatric readmissions were closely related to physical comorbidities (9).

Traditional psychiatric wards increasingly failed to meet the complex treatment needs of these patients, driving the emergence of Psychiatric Intensive Care Units (PICUs). The establishment of PICUs marked a critical step toward the specialization and intensification of care in psychiatric institutions. These units were designed to provide high-level monitoring and treatment for high-risk patients whose psychiatric symptoms were severe or whose physical conditions had acutely worsened. However, such patients often faced difficulty receiving continuous care in general hospitals due to behavioral challenges, while psychiatric hospitals traditionally lacked the capacity to manage severe physical illnesses—leading to repeated transfers, delayed treatment, and poor outcomes.

Against this backdrop, the psychiatric emergency department (PED) increasingly became the first point of entry for these complex cases. A large number of patients presenting with both psychiatric and acute physical conditions entered the treatment system through the PED. Therefore, the emergency department not only managed psychiatric behavioral disturbances but also was required to identify and provide initial intervention for life-threatening physical conditions. Working in close coordination with the PICU, the PED played a vital role in establishing a closed-loop system of “early identification—early intervention—continuous monitoring.” This integrated PED–PICU model facilitated the development of a psychiatric critical care system with distinct disciplinary features. Thus, the integration of psychiatric emergency services with PICUs into a unified acute and critical care unit became an essential strategy for psychiatric hospitals to address the challenges of managing comorbid conditions and enhanced their comprehensive treatment capabilities. This approach also provided a strong foundation for building a modern mental health service system characterized by multidisciplinary collaboration, streamlined processes, and improved care efficiency.

## 2 Integrate process and practice

### 2.1 Establishment and development of the psychiatric intensive care unit (PICU)

Established in 1954, the Mental Health Center of Zhejiang University School of Medicine (Hangzhou Seventh People's Hospital) was a national Class A tertiary psychiatric hospital that integrated medical care, teaching, research, rehabilitation, and prevention. To promote the standardized development of psychiatric intensive care and enhance the hospital's capacity to provide comprehensive treatment for patients with psychiatric disorders complicated by critical physical illnesses, the hospital established the Department of Intensive Care Medicine (ICU) on February 15, 2019. The department primarily admitted patients with severe physical illnesses comorbid with psychiatric disorders, focusing on addressing the challenges in treating psychiatric patients with critical physical conditions and managing psychiatric symptoms secondary to serious somatic or organic diseases.

The design and medical management of the Psychiatric Intensive Care Unit (PICU) strictly adhered to the relevant standards outlined in the Guidelines for the Construction and Management of Intensive Care Units (Trial) issued by China's Ministry of Health {Document No. [(2009)] 23}, and were optimized to accommodate the specific needs of psychiatric care, aiming to meet the specialized and systematic requirements for treating critically ill psychiatric patients. When the Department of Intensive Care Medicine was first established, it was equipped with only two beds. Through continuous expansion, it included 11 standard ICU beds, each occupying approximately 15 square meters, with an average distance of one meter between beds. The ward was outfitted with independent ventilation, disinfection, lighting, and emergency power systems, all in accordance with the standards of general hospital ICUs. Each monitored bed was equipped with a comprehensive vital signs monitoring system capable of continuous tracking of ECG, blood pressure, pulse, and oxygen saturation. The beds supported both basic and advanced life support functions and were equipped with standard emergency devices such as ventilators, infusion pumps, and syringe pumps. Additional equipment in the ward included video laryngoscopes, fiberoptic bronchoscopes, and complete resuscitation facilities, providing full-scale support for patients with severe psychiatric disorders. At that time, the department was equipped with 11 invasive and non-invasive ventilators, 15 ECG monitors, 1 transport ventilator, high-flow oxygen therapy devices, 2 intubation kits, 2 bedside ultrasound machines, 1 defibrillator, 1 electrocardiograph, 1 vibration sputum drainage device, 7 manual resuscitators, 2 fiberoptic bronchoscopes, 2 air disinfection machines, 2 bed unit disinfection machines, 15 infusion pumps, 10 enteral nutrition pumps, 2 micro-infusion hypothermia therapy devices, 11 air mattresses, 2 pneumatic therapy devices, and mobile nursing carts. The central oxygen supply system fully covered the ward, providing robust respiratory support for patients with multisystem dysfunction. In terms of staffing, the physician-to-bed ratio was 0.8:1 and the nurse-to-bed ratio was 2.5:1. The ward included single isolation rooms reserved for infectious disease cases or other special needs, and routinely kept one vacant bed available to respond to emergency admissions. At that time, the department had 9 physicians, including 3 associate chief physicians, 4 attending physicians, and 2 resident physicians. The nursing team comprised 22 members, including 3 associate chief nurses (senior title), 7 supervising nurses (intermediate title), and 12 staff nurses (junior title). Overall, the team possessed solid clinical treatment capabilities and a strong foundation for scientific research.

The Department of Intensive Care Medicine admitted the following types of patients:

• Patients who presented with catatonia or sub-catatonic states accompanied by severe electrolyte imbalance, severe infections, respiratory failure, etc.;
• Patients who had severe psychiatric complications such as malignant catatonia or serotonin syndrome;
• Patients with psychiatric disorders complicated by or secondary to conditions such as diabetic ketoacidosis, hypoglycemic shock, severe infections, heart failure, or respiratory failure;
• Patients with alcohol dependence who developed severe withdrawal syndromes during the detoxification process;
• Patients with delirium that resulted from serious physical illnesses such as pneumonia or encephalitis;
• Patients with severe psychiatric symptoms that led to food refusal, anorexia, or difficulty in eating, resulting in severe hypoalbuminemia, electrolyte imbalance, or infections;
• Patients with acute poisoning caused by psychiatric medications such as clozapine, lithium carbonate, or valproic acid.

Once a patient's condition stabilized, they were transferred back to their original ward for ongoing treatment. Emergency patients admitted to the ICU also were transferred to appropriate psychiatric subspecialty wards for further management after stabilization. Based on ICU admission criteria from general hospitals and considering the specific characteristics of psychiatric patients, the department formulated admission and discharge criteria tailored to the psychiatric ICU setting.

Admission criteria included:

• Acute, reversible, life-threatening organ dysfunction that required intensive monitoring and treatment for short-term recovery;
• Presence of severe risk factors without current organ failure, where ICU management significantly reduced the risk of mortality;
• Acute exacerbation in patients with pre-existing chronic organ dysfunction, with a possibility of returning to baseline function.

Discharge criteria included:

• Acute organ dysfunction was largely corrected, and the patient could be transferred to another department for further treatment;
• The condition had stabilized or entered a chronic phase;
• Patients who were unlikely to benefit from continued intensive care (e.g., those with terminal cachexia or irreversible multiple organ failure) and for whom prolonged ICU stay was not appropriate.

### 2.2 Functional integration of the psychiatric emergency department (PED)

Before the formal establishment of the Department of Intensive Care Medicine, the primary function of the Psychiatric Emergency Department (PED) was limited to managing acute episodes of typical psychiatric symptoms such as suicide attempts and impulsive behaviors. It mainly served as an “access point” for care, with a relatively narrow scope of clinical function. Patients usually were transferred to inpatient wards for further treatment after an initial assessment, as the emergency department lacked the capacity for ongoing treatment and complex medical interventions. However, in recent years, the proportion of psychiatric patients with comorbid physical illnesses rose significantly (3, 6), and the complexity of cases presenting to the emergency department increased, challenging the traditional functional boundaries of psychiatric emergency care. Following the establishment of the ICU, the hospital rapidly promoted the integrated development of the PED and ICU, leading to the formation of a collaborative “Emergency–Critical Care Linkage” model. With the ICU's expertise in managing critical physical conditions, the PED gradually developed the capability to identify and manage severe physical illnesses at an early stage. It no longer simply referred patients with physical complications to general hospitals but instead enabled early recognition, initial intervention, and rapid triage within the psychiatric hospital itself.

Following the integration, the composition of patients in the Psychiatric Emergency Department (PED) underwent a significant transformation. While early emergency admissions primarily were characterized by typical psychiatric and behavioral issues, the patient population gradually evolved to include a substantial number of complex cases involving severe physical comorbidities or organic mental disorders. Some patients requiring further intensive treatment were rapidly transferred to the ICU for continued care after initial stabilization in the emergency department. This shift marked a functional upgrade from “initial triage and referral” to “preliminary management plus high-risk intervention” significantly enhancing the medical service capacity and scope of the emergency department. In parallel, the staffing structure also was adjusted: physicians from the Department of Intensive Care Medicine began rotating through the psychiatric emergency department. These emergency physicians not only gained clinical experience in managing psychiatric symptoms but also developed the ability to identify and initiate treatment for life-threatening physical conditions, resulting in a dual-competency workforce. This initiative improved diagnostic and treatment efficiency and significantly streamlined pre-hospital transfers. Emergency medical systems (e.g., the 120 ambulance service) no longer hesitated to refer patients due to perceived limitations in psychiatric emergency care, thus strengthening the hospital's ability and confidence to admit critically ill psychiatric patients.

### 2.3 Development of specialized centers for rapid response and critical psychiatric care

Following the integration of the Psychiatric Emergency Department and the Department of Intensive Care Medicine, the hospital accumulated extensive clinical experience over the past six years, gradually establishing a specialized diagnostic and treatment system for psychiatric emergencies and critical care. As the patient profile and disease spectrum became clearer, the hospital shifted from a passive reception model to a proactive system of identification and stratified management. This evolution allowed the Psychiatric ICU (PICU) to gradually identify high-incidence and high-severity conditions suitable for specialized treatment, laying the practical foundation and strategic direction for the development of dedicated treatment centers. During the early exploratory phase, the scope of PICU admissions was not clearly defined, and physicians still were developing a concrete understanding of what constituted “critical psychiatric illness.” Through the accumulation of clinical practice, several high-risk disease categories with strong indications for specialized inpatient care were identified, including:

#### Drug intoxication-related conditions

These encompassed an integrated management approach from pre-hospital emergency interventions (e.g., gastric lavage), toxicological assessment, and the recognition and treatment of drug-induced seizures or delirium, to fluid resuscitation and enhanced elimination.

#### Catatonia with multiple somatic complications

For example, catatonia was accompanied by pneumonia, pulmonary embolism, intestinal obstruction, or rhabdomyolysis, which could rapidly progress to life-threatening conditions, making them typical cases for PICU admission.

#### Delirium and severe physical illness in elderly patients with psychiatric disorders

Particularly those with dementia and delirium complicated by sepsis, severe pneumonia, or respiratory failure.

#### Severe alcohol-related withdrawal syndromes

Such as delirium tremens with profound dehydration, electrolyte imbalance, secondary seizures, and malnutrition, which often were marked by volatile clinical courses.

Based on the identification of these high-frequency, high-risk conditions, the hospital established three specialized diagnostic and treatment centers, each with clearly defined functional roles:

#### Complex and critical psychiatric disorders treatment center

This center focused on managing catatonia with severe physical comorbidities and multisystem dysfunctions in psychiatric patients. By integrating resources from the ICU, emergency department, and specialty teams, the center facilitated cross-disciplinary and cross-disease collaboration, significantly enhancing the capacity for early identification and intervention in high-risk cases.

#### Psychiatric drug toxicology treatment center

This center was designed to address common psychiatric scenarios such as intentional overdose or suicide attempts via medication. It developed a closed-loop treatment pathway spanning emergency gastric lavage, toxicology monitoring in the ICU, and rehabilitation assessment. It placed particular emphasis on the early recognition and intervention of drug-induced delirium, seizures, and consciousness disturbances.

#### Rapid antidepressant treatment center

To meet the demand for fast-acting interventions, the hospital pioneered the use of intranasal esketamine in a psychiatric emergency outpatient setting—the first such pathway established in China. The center operated with two dedicated observation beds and two additional backup beds, accommodating up to 4–5 patients simultaneously. To ensure precision and safety, appointments were required for treatment.

As these centers became increasingly standardized and operationally mature, the Psychiatric Emergency Department evolved from a traditional “initial reception” role to a professional closed-loop process of “rapid identification–early intervention–precise triage.” This transformation significantly enhanced the hospital's overall capacity to manage psychiatric emergencies and their comorbid conditions, and laid a critical foundation for developing a subspecialty system centered on psychiatric intensive care.

## 3 Statistical analysis and results

To illustrate the data from the six years following the establishment and integration of the Psychiatric Emergency Department (PED) and the Psychiatric Intensive Care Unit (PICU), we summarized the emergency admissions in the PED and the medical procedures performed in the PICU, and presented the temporal trends. Data from 2019 to 2024 were extracted from Table 1 (procedures) and Table 2 (departmental admissions). The analysis was performed using R software (version 4.5.1; R Foundation for Statistical Computing, Vienna, Austria; available at https://www.r-project.org), with the dplyr, tidyr, and ggplot2 packages for data wrangling and visualization, along with stats for statistical testing. First, we applied the Cochran–Armitage trend test (implemented as prop.trend.test) to assess whether the proportion of PICU procedures or PED admissions exhibited significant monotonic changes over time. Second, we fitted binomial logistic regression models (glm with family = binomial) for each procedure/department, with year as the independent variable and the number of events vs. non-events as the outcome. Odds ratios (OR) per year with 95% confidence intervals (CI) were calculated to quantify the yearly change in odds of undergoing a given procedure or being admitted via PED. Graphical representations (Figuress 1,2) were generated using ggplot2 and ggrepel, where observed proportions were shown as solid lines, and fitted logistic regression trends as dashed lines, annotated with corresponding ORs and *p*-values.

**Table 1:** Comparison of the number and ratio of PICU* and All patients in Various procedures from 2019 to 2024.

Procedure	2019 （PICU/ALL）		2020 （PICU/ALL）		2021 （PICU/ALL）		2022 （PICU/ALL）		2023 （PICU/ALL）		2024 （PICU/ALL）	
	Number	Ratio	Number	Ratio	Number	Ratio	Number	Ratio	Number	Ratio	Number	Ratio
Rescue due to Life-Threatening	10/49	20.40%	11/36	30.60%	16/54	29.60%	37/77	48.10%	60/106	56.60%	37/64	57.80%
Endotracheal Intubation	4/8	50.00%	3/8	37.50%	10/20	50.00%	24/29	82.70%	25/32	78.10%	19/23	82.60%
Central Venous Catheterization	23/27	85.20%	21/26	80.80%	60/63	95.20%	62/68	91.20%	65/71	91.60%	85/88	96.50%
Lumbar Puncture	2/40	5.00%	1/45	2.20%	11/97	11.30%	15/71	21.10%	11/60	18.30%	13/71	18.30%
Thoracentesis	1/1	100.00%	5/5	100.00%	8/11	72.70%	16/16	100.00%	15/19	78.90%	14/16	87.50%
Gastrointestinal Tube	9/49	18.40%	6/528	1.10%	54/477	11.30%	144/511	28.10%	125/375	33.30%	140/311	45.00%

^a^ PICU, Psychiatric Intensive Care Unit.

**Table 2:** Comparison of the number and ratio of inpatient admissions from PED^a^ and All patient admissions from 2019 to 2024.

Department	2019 (PED/ALL)		2020 (PED/ALL)		2021 (PED/ALL)		2022 (PED/ALL)		2023 (PED/ALL)		2024 (PED/ALL)	
	Number	Ratio	Number	Ratio	Number	Ratio	Number	Ratio	Number	Ratio	Number	Ratio
Total	849/21,644	3.92%	2,350/21,597	10.88%	3,679/21,421	17.17%	4,134/20,268	20.39%	4,946/22,430	22.05%	5,456/22,650	24.09%
PICU	1/36	2.78%	1/20	5.00%	101/187	54.01%	202/307	65.80%	248/396	62.63%	309/434	71.20%
Geriatric	63/5,647	1.12%	232/6,182	3.75%	392/5,659	6.93%	463/5,005	9.25%	503/4,461	11.28%	498/3,283	15.17%
Child Psychiatry	2/883	0.23%	25/846	2.96%	36/1,020	3.53%	39/978	3.99%	32/1,008	3.17%	52/1,361	3.82%
General Psychiatry	666/8,808	7.56%	1,597/8,832	18.08%	2,396/8,127	29.48%	2,627/7,906	33.23%	2,937/8,000	36.71%	3,292/7,186	45.81%
Psychosomatic Medicine	40/5,688	0.70%	251/5,206	4.82%	382/5,880	6.50%	414/5,668	7.30%	669/8,005	8.36%	956/10,036	9.53%
Homeless Patients without Primary Care	77/582	13.23%	244/511	47.75%	372/548	67.88%	389/404	96.29%	557/560	99.46%	349/350	99.71%

^a^ PED, Psychiatric Emergency Department.

**Figure 1:**
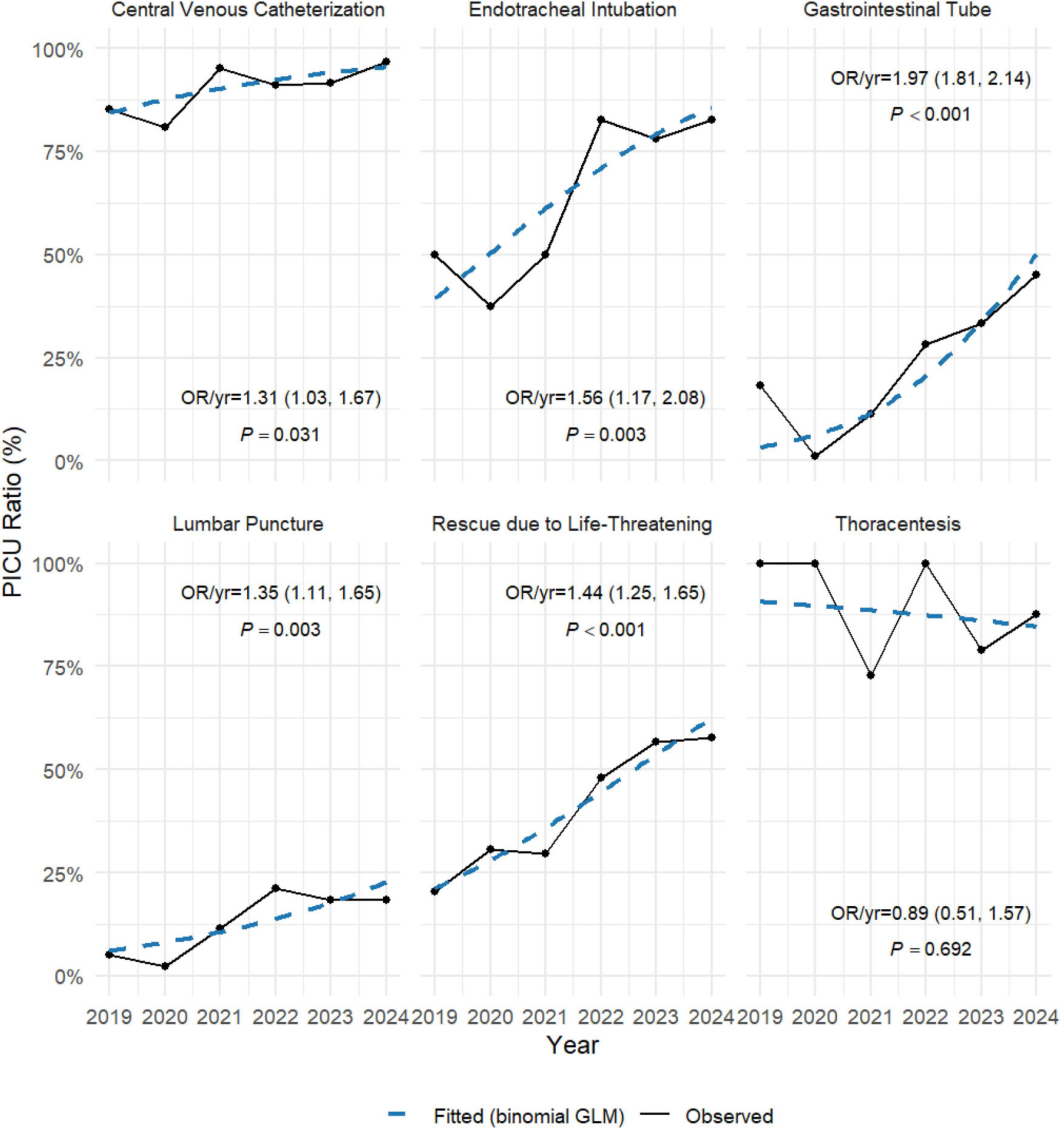
PICU vs. ALL: Procedure ratio trends (2019-2024).

**Figure 2:**
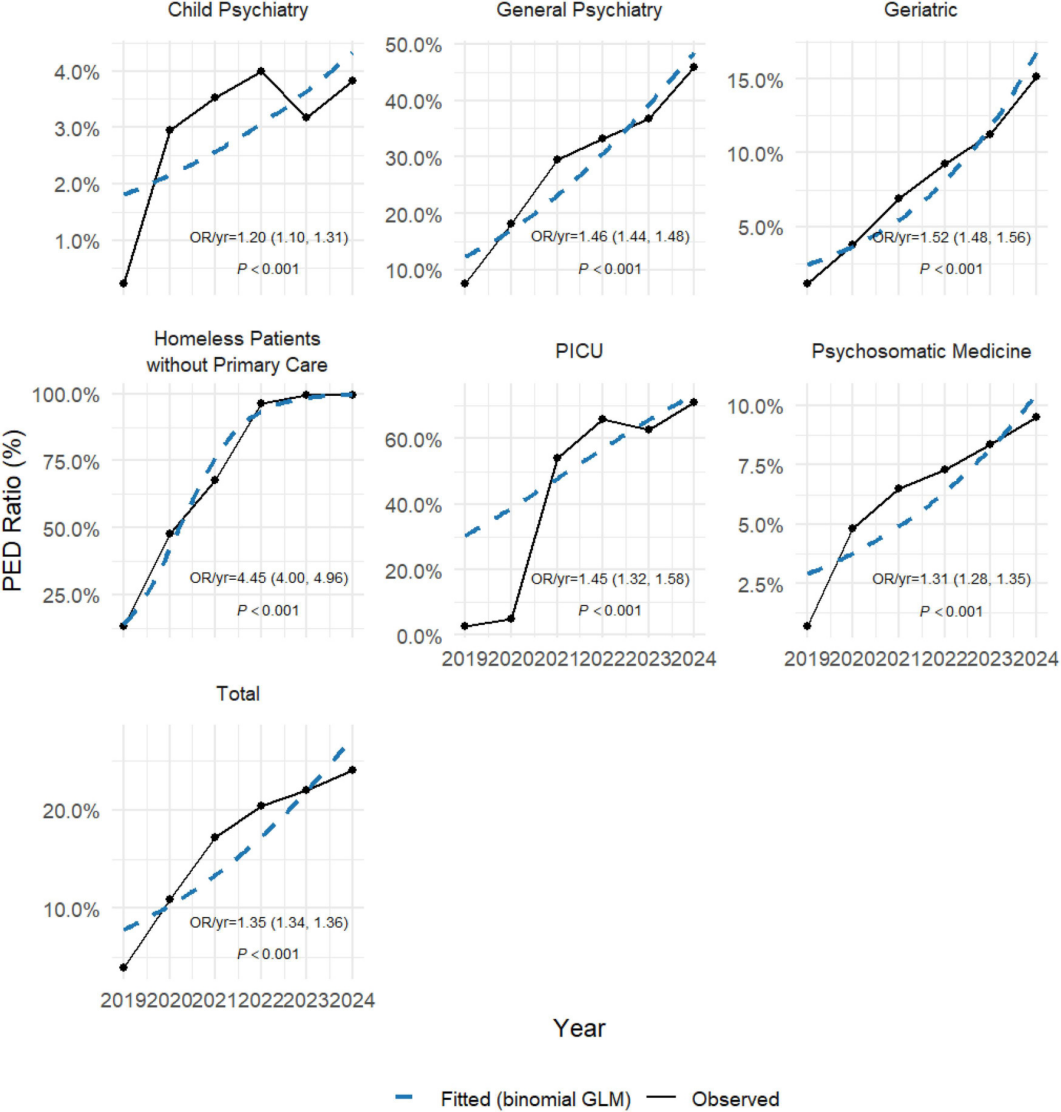
PED/ALL ratio by department (2019-2024).

Table 1 presented a comparison of the number and ratio of patients in the Psychiatric Intensive Care Unit (PICU) and all patients in the hospital in terms of various medical procedures from 2019 to 2024. The data included life-saving interventions as well as several invasive procedures, such as endotracheal intubation, central venous catheterization, lumbar puncture, thoracentesis, and gastrointestinal tube insertion, reflecting the annual changes in these medical procedures. The table showed a gradually increasing ratio of patients in the PICU receiving these procedures, highlighting the progressive development of the PICU over time (Table 1). Figure 1 illustrated the trends in PICU procedures relative to all hospitalized patients from 2019 to 2024. The ratios of invasive interventions such as endotracheal intubation, central venous catheterization, and gastrointestinal tube insertion increased significantly over time. Logistic regression analysis confirmed significant upward trends (e.g., endotracheal intubation: OR/year = 1.97, 95% CI: 1.81–2.14, *p* < 0.001; central venous catheterization: OR/year = 1.31, 95% CI: 1.03–1.67, *p* = 0.031), indicating a progressive enhancement of PICU procedural capacity.

Table 2 presented a comparison of the number and ratio of patients admitted from the Psychiatric Emergency Department (PED) and all patient admissions to different departments in the hospital from 2019 to 2024. The data showed that the proportion of admissions from the PED steadily increased, particularly the significant rise in the ratio of admissions to the PICU. This change reflected the increasing role of the PED in managing psychiatric patients with severe comorbid physical conditions, as the PED handled more patients with serious physical diseases (Table 2). Figure 2 presented the annual proportion of admissions from the Psychiatric Emergency Department (PED) across hospital departments. The overall ratio of PED-derived admissions rose steadily (OR/year = 1.35, 95% CI: 1.34–1.36, *p* < 0.001). Notably, admissions to the PICU via PED increased most markedly (OR/year = 1.45, 95% CI: 1.32–1.58, *p* < 0.001), reflecting the growing role of the PED–PICU integrated model in managing complex psychiatric emergencies with comorbid physical illness.

**Figure 3:**
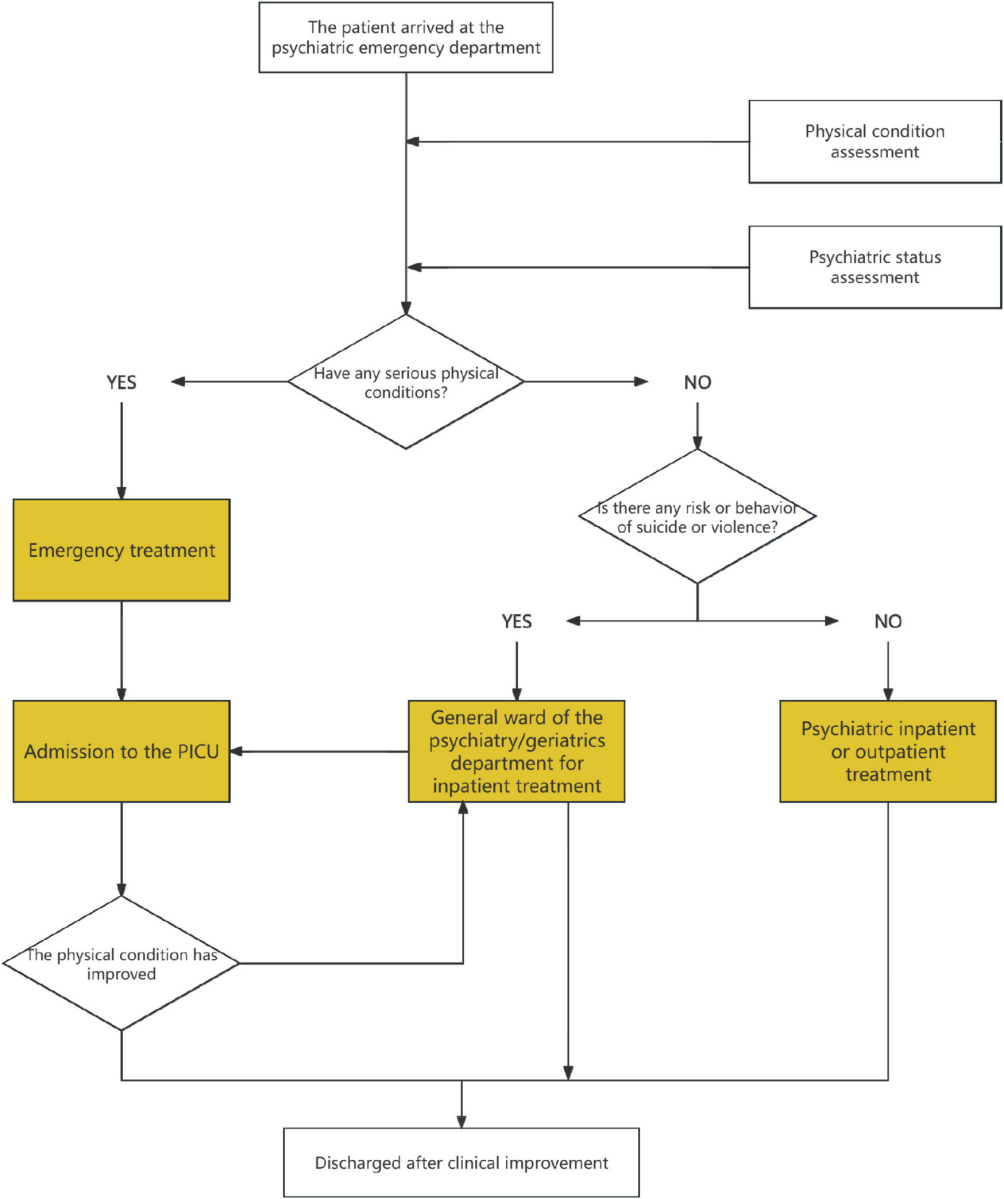
Integrated pathway and closed-loop management model between psychiatric emergency department (PED) and psychiatric intensive care unit (PICU).

Figure 3 illustrated the integrated care pathway between the Psychiatric Emergency Department (PED) and the Psychiatric Intensive Care Unit (PICU). Upon arrival at the PED, patients underwent simultaneous assessments of their physical and psychiatric status. If a severe physical condition was identified, emergency treatment was initiated, and the patient subsequently was admitted to the PICU for continuous intensive care. After stabilization of the acute medical condition, patients were transferred to general psychiatric or geriatric wards for further management until clinical stabilization was achieved. Conversely, if no critical physical illness was present, triage focused on assessing the risk of suicide or violence: patients with such risks were admitted to general psychiatric wards, while those without were directed to psychiatric inpatient or outpatient treatment. In addition, for psychiatric inpatients who developed serious physical illnesses during hospitalization, transfer to the PICU also was possible for further intensive care, with subsequent return to psychiatric wards once the physical condition improved. Ultimately, all patients were discharged after clinical improvement. This closed-loop pathway highlighted the central role of the PED–PICU integrated model in enabling early recognition, timely intervention, and continuous care for psychiatric patients with comorbid critical medical conditions, thereby strengthening the hospital's capacity for comprehensive management.

## 4 Formation and influence of the psychiatric emergency and critical care subspecialty

Based on the simultaneous development and deep integration of the Psychiatric Intensive Care Unit (PICU) and the Psychiatric Emergency Department (PED), the hospital's emergency and critical care system evolved from nonexistence to a structured and standardized framework. It was through this process that psychiatric emergency and critical care medicine gradually took shape as an independent clinical subspecialty and continued to mature and improve. It was important to emphasize that the formation of this subspecialty was not a simple replication or replacement of emergency medicine or EICU models found in general hospitals. The goal was not centered on general ICU technologies such as cardiopulmonary resuscitation, ECMO, blood purification, or infection control, nor did we aim to manage all categories of multi-organ failure. Instead, we focused on building an emergency–critical care system with distinct psychiatric characteristics, specifically targeting acute and critical conditions that were unique to psychiatric disorders, were caused by psychiatric symptoms, or were primarily manifested through psychiatric symptoms.

Patients with psychiatric emergencies and critical conditions often developed severe physical complications secondary to abnormal behaviors such as food refusal, catatonia, or agitation, or due to psychiatric medication overdose and self-harm. These complications included electrolyte imbalances, pneumonia, myocardial injury, venous thromboembolism (VTE), and acute drug intoxication (5, 8). At the same time, certain primary organic neurological diseases—such as encephalitis or autoimmune encephalopathies—sometimes initially presented with psychiatric symptoms, which made them highly susceptible to misdiagnosis or delayed diagnosis in clinical practice. Under traditional healthcare systems, such patients frequently were transferred back and forth between psychiatric hospitals and general hospitals, which increased the burden on patients and their families and delayed timely treatment. In response, our hospital proposed and actively implemented the management principle of ‘’not allowing patients to shuttle between two systems’’ By leveraging the PICU platform, patients received essential interventions—such as respiratory support, lumbar puncture, vital signs monitoring, gastric lavage, detoxification, and nutritional support—within the psychiatric specialty hospital itself. This enabled integrated, closed-loop management of psychiatric and physical comorbidities, and significantly improved treatment efficiency and continuity.

In developing this subspecialty, we recognized that the rapid accumulation of high-quality cases and the establishment of a scientific operational framework were key to the growth of any emerging discipline. As psychiatric PICUs remained in an exploratory phase nationwide, with few mature models to draw upon, our hospital faced early skepticism—particularly concerns about high construction costs paired with limited short-term recognition or return. In response, we took the lead in implementing an integrated emergency–critical care management model, in which intensive care physicians directly participated in emergency consultations, thereby establishing a psychiatric specialty coordination system modeled on the structure of an EICU. This model led to three significant changes. First, the primary source of admissions shifted from internal department referrals to direct emergency admissions, which greatly improved the efficiency of initial consultations and increased the clinical complexity of cases. Second, the disease spectrum specific to psychiatric critical care rapidly was clarified and stabilized, and now encompassed catatonia/sub-catatonia with infection and metabolic disturbances, malignant syndrome, serotonin syndrome, delirium tremens, and dementia with delirium in elderly patients. Third, ICU admission and discharge criteria tailored to the characteristics of psychiatric medicine were established, clearly defining the boundaries for admission as patients with a psychiatric disorder, an acute life-threatening physical condition, and limited potential for referral to general hospitals. These developments promoted the refinement and institutionalization of the psychiatric critical care system.

Through institutional innovation and continuous clinical advancement, our department steadily increased its recognition and influence both within and beyond the hospital. In 2024, the hospital took the lead in establishing the “Critical Care Subgroup of the Psychiatric Specialty Alliance of the Mental Health Center, Zhejiang University,” in collaboration with 17 psychiatric specialty hospitals across China that possessed emergency and critical care capabilities. This initiative marked a significant enhancement of our organizational capacity and leadership in the field. In the same year, we successfully hosted the national continuing medical education (CME) program titled “Diagnosis, Treatment, and Latest Advances in Psychiatric Emergencies and Critical Care”, which promoted standardized training and dissemination of psychiatric critical care concepts, and effectively expanded our academic influence nationwide.

## 5 Discussion and prospects

Although intensive care medicine was well established in general hospitals and was recognized as a benchmark of institutional capacity, the establishment of ICU departments within psychiatric specialty hospitals remained relatively new. In particular, psychiatric ICUs that focused on managing psychiatric disorders with comorbid critical physical illnesses still were in the early stages of exploration. Since its inception, our department upheld the philosophy of “acknowledging medical limitations while striving to overcome them” aiming to transform the traditional model from “passive referral” to “active management” and to provide continuous, efficient, and safe care for critically ill psychiatric patients.

By that time, our team had managed over a thousand severe cases involving intubation, severe infections, and drug intoxication, accumulating substantial cross-disciplinary experience. As one of the first psychiatric hospitals in China to establish a dedicated ICU, we not only filled a professional gap but also set a practical model for others. With advanced equipment and a specialized team, we were fully capable of treating complex psychiatric emergencies.

Beyond clinical care, the PICU also evolved into a platform for research and education. The field demanded professionals who combined general ICU competencies with psychiatric expertise. To address this, we developed a structured training system focused on emergency and critical care in psychiatry, enhancing clinicians' ability to respond to crises such as suicide attempts, altered consciousness, and severe drug reactions.

To date, neither in China nor abroad has a formal PED–PICU integrated model been established in psychiatric hospitals.However, valuable experience could be drawn from the Emergency Department–ICU (ED–ICU or EICU) integration models in general hospitals, which provided a framework for managing critically ill patients at the interface of emergency and intensive care. In the United States, the “Early Intervention Team” (EIT) was established to deliver ICU-level consultation and early management within the ED, bridging the gap before ICU admission (10). Other evolving strategies addressed the challenge of ICU boarders, including temporary ED-based ICUs and expanded resuscitation areas (11). In Europe, similar approaches were implemented, such as the Resuscitative Care Unit in Greece (12) and the Critical Emergency Medicine Unit in Italy (13), both designed to provide immediate intensive care within the ED setting and mitigate delays in treatment. These international experiences highlighted the feasibility and potential benefits of ED–ICU integration in general hospitals, which served as a reference for future development of psychiatric-specific PED–PICU models.

Beyond feasibility, it was acknowledged that direct evidence and practical experience for PED–PICU integration in psychiatric hospitals were still lacking. Nevertheless, findings from general hospital ED–ICU models provided important insights into their potential value. Studies from the U.S. demonstrated that ED-ICU implementation was associated with improved hospital outcomes and more rational ICU utilization (14), with additional evidence of favorable cost-effectiveness (15). Expert commentaries emphasized that ED-ICUs added unique value to healthcare systems by reducing ICU boarding and enabling early interventions (16). Furthermore, patients admitted through ED-ICUs experienced shorter ED lengths of stay and more streamlined care pathways (17). Conversely, prolonged ICU boarding in the ED correlated with worse outcomes, including longer ICU stays and higher mortality (18). Taken together, these results from general hospitals suggested that similar integrated approaches, if adapted to psychiatric emergency and critical care settings, might enhance patient outcomes and optimize resource allocation in psychiatric institutions.

In research, we focused on the interaction between psychiatric and somatic systems. The main directions included:

• Pathophysiological mechanisms in psychiatric critical illness (e.g., coagulopathy, immune activation);
• Early recognition of organic causes of psychiatric symptoms (e.g., autoimmune encephalitis, thyroid dysfunction);
• Development of diagnostic and treatment protocols for conditions like catatonia, delirium, and eating disorders;
• Emergency cohort studies on suicidal and non-suicidal self-injury to build a scientifically grounded, evidence-based care system.

Looking ahead, the PICU of Hangzhou Seventh People's Hospital continued to deepen subspecialty development, enhanced its clinical and academic impact, and strengthened collaboration with general and psychiatric hospitals. We refined referral systems, built a closed-loop management model for psychiatric patients with critical medical conditions, and promoted the integrated development of clinical care, research, and education. Though still an emerging field, psychiatric intensive care had vast potential and played an increasingly vital role in supporting the broader psychiatric care system in China.

## 6 Limitations

This report also had several limitations. First, it represented the experience of a single psychiatric specialty hospital, and the organizational, structural, and regional characteristics of our institution might not have been directly comparable to those of other centers. Second, the model we described was practice-based and primarily presented in a narrative manner, without systematic prospective evaluation or external validation. Third, the absence of parallel examples or comparative frameworks in the existing literature made it difficult to place our experience within a broader evidence base. Finally, while the PED–PICU integrated model appeared to offer practical benefits in our setting, its generalizability remained uncertain, and further validation in other psychiatric hospitals and health systems was warranted.

## 7 Conclusion

The integrated Psychiatric Emergency Department (PED)–Psychiatric Intensive Care Unit (PICU) model in our hospital demonstrated that psychiatric specialty hospitals could independently provide continuous care for patients with severe mental illness complicated by critical physical conditions. By bridging the gap between emergency stabilization and intensive care, the model formed a closed-loop system that ensured timely recognition, rapid intervention, and ongoing treatment. It improved efficiency, reduced unnecessary transfers, strengthened multidisciplinary collaboration, and fostered the emergence of psychiatric critical care as a distinct subspecialty. Although further validation was needed, this pioneering practice offered a valuable reference for advancing psychiatric critical care systems and improving mental health services.

### Definitions of key terms

To ensure clarity and consistency, the following key terms were defined as used in this study:

#### Psychiatric intensive care unit (PICU)

In this article, PICU referred to a specialized intensive care unit within a psychiatric hospital that was dedicated to managing patients with psychiatric disorders complicated by severe or life-threatening physical illnesses. Unlike general hospital ICUs, the PICU operated under a psychiatric framework while providing continuous somatic life-support and intensive monitoring.

#### Emergency–critical care linkage

This term described the integrated model that directly connected the Psychiatric Emergency Department (PED) with the PICU, enabling seamless transition from emergency triage and stabilization to intensive care management. The linkage emphasized early identification of critical physical illnesses in psychiatric patients, rapid intervention, and timely transfer to the PICU when necessary.

#### Closed-loop system

The closed-loop system referred to a structured care pathway in which psychiatric patients with acute or critical comorbidities were managed through a continuous cycle of emergency assessment, stabilization, intensive care when required, subsequent transfer to general psychiatric wards after improvement, and eventual discharge. This design minimized care gaps and ensured comprehensive, uninterrupted management.
